# Intrauterine Growth Restriction: Cytokine Profiles of Trophoblast Antigen-Stimulated Maternal Lymphocytes

**DOI:** 10.1155/2012/734865

**Published:** 2011-10-31

**Authors:** Raj Raghupathy, Majedah Al-Azemi, Fawaz Azizieh

**Affiliations:** ^1^Department of Microbiology, Faculty of Medicine, Kuwait University, P.O. Box 24923, Kuwait 13110, Kuwait; ^2^Department of Obstetrics & Gynecology, Faculty of Medicine, Kuwait University, P.O. Box 24923, Kuwait 13110, Kuwait; ^3^Department of Mathematics & Biology, Gulf University for Science and Technology, Mubarak Al-Abdullah Area, West Mishref, Hawalli 32093, Kuwait

## Abstract

Intrauterine growth restriction (IUGR) is an important perinatal syndrome that poses several serious short- and long-term effects. We studied cytokine production by maternal peripheral blood lymphocytes stimulated by trophoblast antigens. 36 women with a diagnosis of IUGR and 22 healthy women with normal fetal growth were inducted. Peripheral blood mononuclear cells were stimulated with trophoblast antigens and levels of the proinflammatory cytokines IL-6, IL-8, IL-12, IL-23, IFN**γ**, and TNF**α** and the anti-inflammatory cytokines IL-4, IL-10, and IL-13 were measured in culture supernatants by ELISA. IL-8 was produced at higher levels by blood cells of the IUGR group than normal pregnant women, while IL-13 was produced at lower levels. IL-8, IFN**γ**, and TNF**α** were higher in IUGR with placental insufficiency than in normal pregnancy. IL-12 levels were higher and IL-10 levels were lower in IUGR with placental insufficiency than in IUGR without placental insufficiency. We suggest that a stronger pro-inflammatory bias exists in IUGR as compared to normal pregnancy and in IUGR with placental insufficiency when compared to IUGR without placental insufficiency. Several ratios of proinflammatory to anti-inflammatory cytokines also support the existence of an inflammatory bias in IUGR.

## 1. Introduction

Intrauterine growth restriction (IUGR) is one of the most important perinatal syndromes and is a worldwide problem. IUGR, defined as fetal growth less than the 10th percentile for gestational age [1], puts the fetus and neonate at higher risk for perinatal mortality and morbidity [2] and the child at a permanent risk for a range of disorders that include cardiovascular and renal disease, and hypertension [3]. Affected babies have a 30–50% likelihood of intrapartum hypoxic distress and a 50% risk of neonatal complications that include hypoglycemia, meconium aspiration pneumonia, and long-term growth impairment [4]. 

Intrauterine growth restriction is segregated into two types, IUGR *with* placental insufficiency (or asymmetric IUGR) and IUGR *without* placental insufficiency (or symmetric IUGR). IUGR without placental insufficiency is believed to be an early embryonic event, is constitutional, and is generally attributable to genetic and chromosomal abnormalities, fetal malformation, and infections. Infants of such pregnancies have both length and weight below normal for gestational age; placentas are usually small by weight, but have no other pathologies [5]. On the other hand, IUGR with placental insufficiency (asymmetric IUGR) occurs later in gestation and usually involves a more severe growth restriction of the abdomen than of the head [6]; such pregnancies usually have significant placental pathological findings. IUGR with placental insufficiency is believed to be due to maternal diseases that bring about a reduction of uteroplacental blood flow [6]. 

Despite the delineation of several of the causes and risk factors of IUGR (5–20% due to chromosomal abnormalities, 5–20% due to maternal and fetal vascular disorders and infections [6]), a definite cause of IUGR is not identified in 40–50% of all cases [7]. Logically an insufficient blood flow to the placenta is the first abnormality to suspect and indeed a significant proportion of IUGR cases is associated with placental findings, pointing to problems in fetoplacental circulation [8]. Indeed, the lack of sufficient transport of nutrients and oxygen to the fetus is commonly recognized as leading to IUGR [8], but in a number of cases restricted growth cannot be explained by placental insufficiency alone [8]. In addition to the genetic and constitutional disorders mentioned above, it is appropriate to look at possible immunologic events that may lead to IUGR with and without placental sufficiency. 

Maternal immunologic factors such as cytokines, natural killer (NK) cells, activated macrophages, and lymphocytes have been shown to be associated with several pregnancy complications such as recurrent spontaneous miscarriage, preeclampsia, and preterm delivery. Cytokines have been shown to play vital roles in normal pregnancy both in the maintenance of placental growth and in the modulation of maternal immune reactivity to prevent rejection of the conceptus [9, 10]. The maternal immunologic state that is most conducive to successful pregnancy is maintained by local secretion of T helper-2 (Th2) cytokines and some types of pregnancy complications seem to be associated with a predominance of T helper-1 (Th1) reactivity in the mother; this appears to be the case for recurrent spontaneous miscarriage [11–13], preterm delivery [14, 15], and preeclampsia [16, 17]. 

Th1 and Th2 cells are two of the major subsets of CD4^+^ T-helper cells; they have different cytokine production profiles and accordingly different roles in immune responses. Th1 cells secrete the proinflammatory cytokines IL-2, IFN*γ*, TNF*α*, and TNF*β* which activate macrophages and cell-mediated reactions relevant to cytotoxic reactions and delayed-type hypersensitivity [18, 19]. Th2 cells secrete IL-4, IL-5, IL-10, and IL-13 which induce vigorous humoral immunity [18, 19]. Th1 cytokines tend to be inflammatory cytokines, while some of the Th2 cytokines tend to have anti-inflammatory properties.

While there are numerous studies on cytokine profiles in pregnancy complications like recurrent miscarriage, preterm delivery, and pre-eclampsia [9–17, 20–22], immunological studies in IUGR are relatively small in number. There are few reports on cytokine levels in IUGR. Some studies have estimated cytokines in serum and amniotic fluid, but none have yet focused on cytokine production by maternal lymphocytes. We stimulated maternal peripheral blood mononuclear cells from IUGR pregnancies and normal pregnancies with a trophoblast antigen extract and examined the resulting cytokine production pattern to explore possible relationships between cytokines and IUGR with placental insufficiency and without placental insufficiency.

## 2. Materials and Methods

### 2.1. Subjects

This study has the approval of the Ethics Committee of the Faculty of Medicine, Kuwait University; healthy pregnant women (controls) and subjects with IUGR were inducted into this study after informed consent was obtained from them. Subjects were enrolled at two high-risk pregnancy clinics at Kuwait Maternity Hospital, a tertiary center. Consecutive cases with IUGR were enrolled into the study. All subjects gave informed consent. This prospective study included 36 women with a diagnosis of IUGR and 22 control healthy women with normal fetal growth attending the antenatal clinic at Kuwait Maternity Hospital (Table 1). Power analysis, conducted using the G*Power statistics program (http://www.psycho.uni-duesseldorf.de/abteilungen/aap/gpower3) [23] based on median levels of cytokines measured in our previous studies on cytokines in pregnancy [13, 17, 21], indicated that these sample numbers are adequate to demonstrate differences at the 95% confidence interval. 

Early ultrasound scan was conducted on all subjects to confirm gestational age; inclusion criteria for the IUGR group were fetuses with less than 10th centile abdominal circumference. The 36 women in the IUGR group were further subdivided into 19 IUGR pregnancies with placental insufficiency and 17 IUGR pregnancies without placental insufficiency by assessment of fetal anatomy and biometry, amniotic fluid dynamics, uterine, umbilical, and fetal middle cerebral artery Doppler. Blood velocity waveforms from both uterine arteries, the umbilical artery and the fetal middle cerebral artery, were measured using duplex pulsed-wave Doppler Ultrasound Scanner (ALOKA SSD-650) with 3.5-MHZ convex transducer. Pulsatility Index was calculated as (Systolic/Diastolic)/Systolic as described in [24]. Placental insufficiency was diagnosed if pulsatility index in the umbilical artery was raised, with either absent or reversed end diastolic flow. Doppler measurements were performed by a single investigator.

The control group consisted of 22 women who had a history of at least two previous successful pregnancies with no previous spontaneous miscarriage, pre-eclampsia, preterm labor or IUGR.

### 2.2. Isolation of Peripheral Blood Mononuclear Cells

Five mL of venous blood samples were taken from all subjects within 24 hours of delivery. Peripheral blood mononuclear cells (PBMC) were separated from the blood samples by Ficoll-Hypaque (GE Healthcare, Uppsala, Sweden) density gradient centrifugation, suspended in RPMI medium (GIBCO, Auckland, New Zealand) containing 10% fetal calf serum, aliquoted into 96-well tissue culture plates at a density of 10^5^ cells per well and then challenged with the trophoblast antigen extract as described below.

### 2.3. Trophoblast Antigen Stimulation of PBMC

Trophoblast antigen extracts were prepared as described previously [20–22] from the human gestational choriocarcinoma cell line JEG-3 (American Type Culture Collection, Md, USA), which is of trophoblastic origin. JEG cells were cultured in RPMI-1640 medium until 80% confluence is reached, harvested without trypsinization using a rubber cell scraper, washed three times in medium and then disrupted in a Dounce homogenizer (*∼*100 strokes). The suspension was then centrifuged at 3000 rpm for 10 minutes, the supernatant filtered through a 0.20 *μ*M filter, aliquoted and stored at −20°C until use. This material was used to stimulate maternal peripheral blood cells. Maternal PBMCs were stimulated at a density of 10^5^ cells per well with JEG antigen. Initial standardization experiments in our laboratory (data not shown) showed that the optimal concentration for cell proliferation upon stimulation was 30 *μ*g/mL. PBMCs were cultured for 4 days after antigen stimulation, after which supernatants were collected for cytokine estimation.

### 2.4. Determination of Cytokine Levels by ELISA

Levels of the proinflammatory cytokines IL-6, IL-8, IL-12, IL-23, IFN*γ*, and TNF*α* and the anti-inflammatory cytokines IL-4, IL-10 and IL-13 in trophoblast antigen-stimulated cell culture supernatants were measured by ELISA. Kits for estimating IL-4, IL-8, IL-10, IL-12, IFN*γ* and TNF*α* were obtained from Beckman-Coulter (Marseilles, France), IL-13 kits from R & D Systems (Minneapolis, Minn, USA) and IL-23 kits from Bender Medsystems (Vienna, Austria). Sensitivities of the kits and the reproducibilities within and between assays are provided in the appendix below. The manufacturer's protocols were followed for these assays which are based on the antibody sandwich principle. Samples were tested in triplicate and absorbance values read using an ELISA Reader. Accurate sample concentrations of cytokines were determined by comparing their respective absorbancies with those obtained for the reference standards plotted on a standard curve.

### 2.5. Statistical Analyses

The standard Mann-Whitney-*U* test was used for nonparametric comparisons of median cytokine levels, as the data were not normally distributed. Differences were considered significant if the *P* value ≤0.05.

## 3. Results

We stimulated maternal PBMC with the trophoblast antigen extract and then measured the levels of the proinflammatory cytokines IL-6, IL-8, IL-12, IL-23, IFN*γ* and TNF*α* and the anti-inflammatory cytokines IL-4, IL-10 and IL-13. Median levels of cytokines were compared for statistical significance. We also calculated the means of ratios of proinflammatory to anti-inflammatory cytokines (e.g., IFN*γ*/IL-4, IL-8/IL-10). This was done to determine whether bias or dominance of pro- or anti-inflammatory cytokines exists in the stimulated cultures. The following groups were compared statistically: IUGR versus normal pregnancy, IUGR with placental insufficiency versus normal pregnancy, IUGR without placental insufficiency versus normal pregnancy and finally IUGR with placental insufficiency versus IUGR without placental insufficiency.

### 3.1. Comparison of Cytokine Profiles in IUGR versus Normal Pregnancy

**Figure 1:**
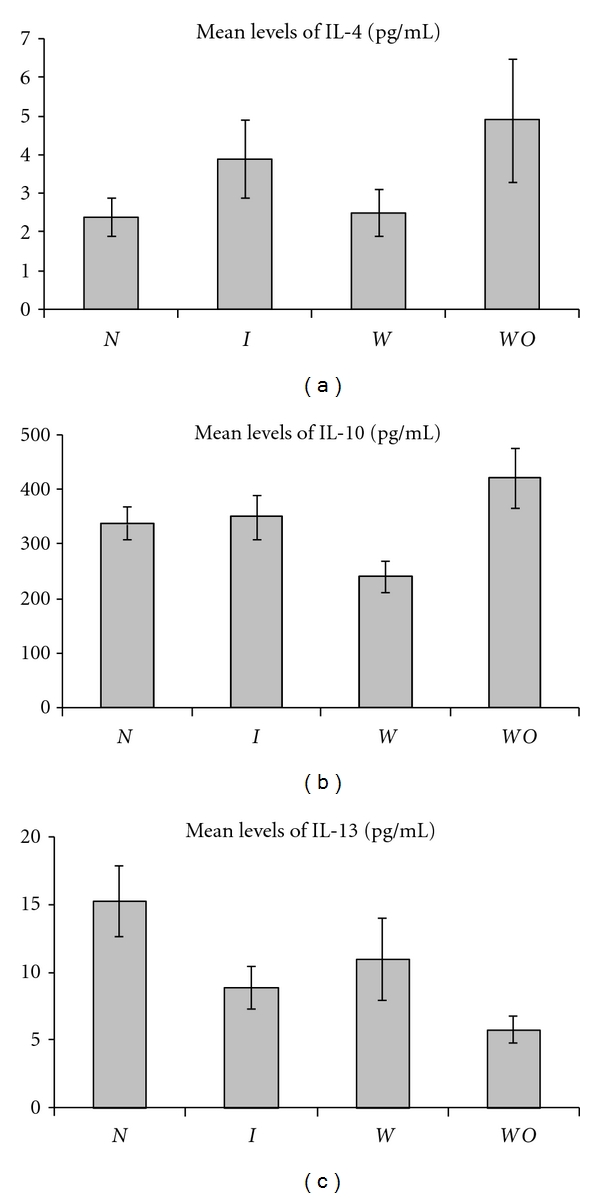
Mean levels of the anti-inflammatory cytokines IL-4, IL-10, and IL-13 produced by PBMC from normal pregnancy (*N*), all IUGR subjects (*I*), IUGR with placental insufficiency (*W*), and IUGR without placental insufficiency (*WO*).

**Figure 2:**
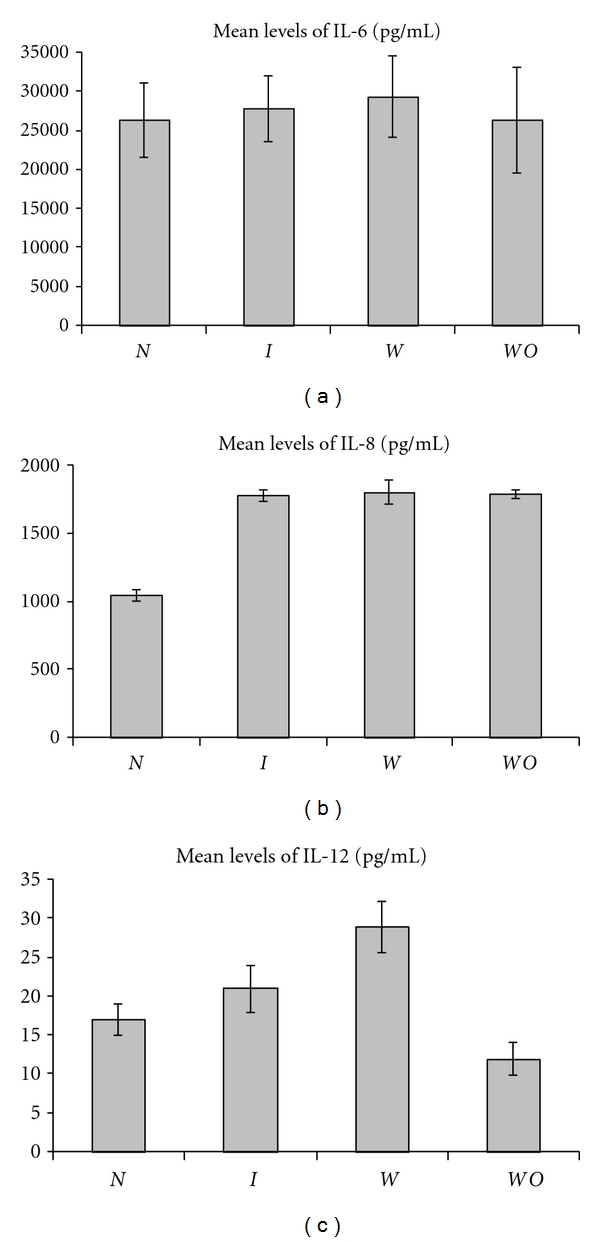
Mean levels of the proinflammatory cytokines IL-6, IL-8, and IL-12 produced by PBMC from normal pregnancy (*N*), all IUGR subjects (*I*), IUGR with placental insufficiency (*W*), and IUGR without placental insufficiency (*WO*).

We found significantly higher levels of the proinflammatory cytokine IL-8 (mean ± SEM = 1780 pg/mL ± 44) in IUGR (i.e., all IUGR pregnancies) as compared to normal pregnancy (mean ± SEM = 1049 pg/mL ± 45) (*P* < 0.0001) (Figure 2). We also found significantly lower levels of the anti-inflammatory cytokine IL-13 in IUGR (8.9 pg/mL ± 1.6) versus normal pregnancy (15.3 pg/mL ± 2.6) (*P* < 0.02) (Figure 1). The IL-8/IL-13 ratio is also higher in IUGR as compared to normal pregnancy (*P* < 0.0005). Other cytokine ratios which are significantly higher in IUGR than in normal pregnancy are IL-12/IL-13 (*P *< 0.02), IL-6/IL-13 (*P* < 0.01) and TNF*α/*IL-13 (*P* < 0.02) (Table 2). Other cytokine ratios were not significantly different between IUGR and normal pregnancy. Based on the higher levels of IL-8, the lower levels of IL-13 and the higher mean cytokine ratios mentioned above, we suggest that a proinflammatory cytokine pattern exists among PBMC from IUGR subjects. However, we found higher levels of the proinflammatory cytokine IL-23 in normal pregnancy (479 pg/mL ± 15) than in IUGR (356 pg/mL ± 13) (*P* < 0.0001). The IL-23/IL-4 (*P* < 0.003) and IL-23/IL-10 (*P* < 0.005) ratios are also higher in IUGR versus normal pregnancy.

### 3.2. Comparison of Cytokine Profiles in IUGR with Placental Insufficiency versus Normal Pregnancy

**Figure 3:**
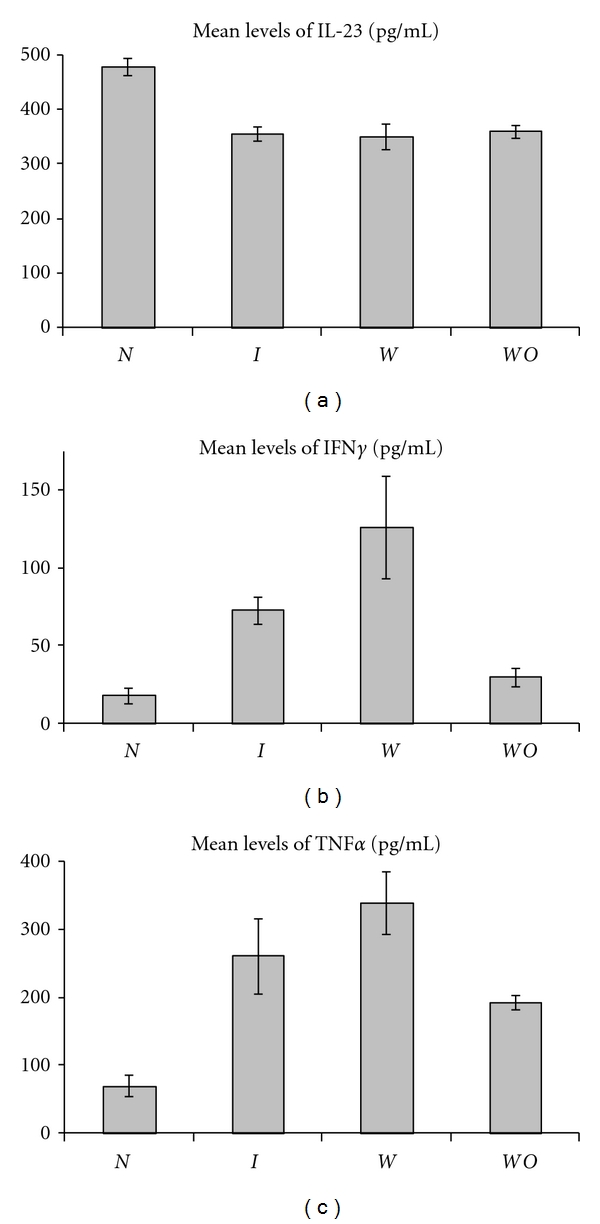
Mean levels of the proinflammatory cytokines IL-23, IFN*γ*, and TNF*α* produced by PBMC from normal pregnancy (*N*), all IUGR subjects (*I*), IUGR with placental insufficiency (*W*), and IUGR without placental insufficiency (*WO*).

The levels of the proinflammatory cytokines IL-8 (1803 pg/mL ± 89, *P* < 0.001), IFN*γ* (126 pg/mL ± 33, *P* < 0.02), and TNF*α* (340 pg/mL ± 46, *P* < 0.04) are significantly higher in IUGR with placental insufficiency as compared to normal pregnancy (1049 pg/mL ± 45, 18 pg/mL ± 6, 70 pg/mL ± 21, resp.). The IL-12/IL-13 and IL-12/IL-10 ratios are significantly higher in IUGR with placental insufficiency when compared to normal pregnancy (*P* < 0.04 in both cases) (Table 2). The higher ratios and the higher levels of IL-8, IFN*γ*, and TNF*α* are suggestive of a higher proinflammatory bias in IUGR with placental insufficiency than in normal pregnancy. None of the other cytokines, except for IL-23 (Figure 3), and none of the other ratios, except for IL-23/IL-4 were significantly different; IL-23 levels were significantly higher in normal pregnancy (479 pg/mL ± 15) versus IUGR with placental insufficiency (350 pg/mL ± 24) (*P* < 0.0001) and the IL-23/IL-4 ratio was also higher in normal pregnancy (*P* < 0.01).

### 3.3. Comparison of Cytokine Profiles in IUGR without Placental Insufficiency versus Normal Pregnancy

The proinflammatory cytokine IL-8 is produced at higher levels by PBMC from IUGR without placental insufficiency (1793 pg/mL ± 33) than by PBMC from normal pregnant controls (1049 pg/mL ± 45) (*P* < 0.0001). On the other hand, the anti-inflammatory cytokine IL-13 is produced at lower levels by PBMC from IUGR without placental insufficiency (5.8 pg/mL ± 1) than by PBMC from normal pregnant controls (15.3 pg/mL ± 2.6) (*P* < 0.002) (Figures 1 and 2). Two of the ratios, IL-6/IL-13 (*P* < 0.006) and IL-8/IL-13 (*P* < 0.001), were significantly higher in IUGR without placental insufficiency compared to normal pregnancy. However, the IFN*γ*/IL-10 ratio was actually higher in normal pregnancy than in IUGR without placental insufficiency (*P* < 0.03) (Table 2). The higher IL-8 levels and the lower IL-13 levels suggest that there appears to be a shift towards a proinflammatory bias. As in the two comparisons mentioned above, IL-23 levels were significantly higher in normal pregnancy (479 pg/mL ± 15) than in IUGR without placental insufficiency (361 pg/mL ± 13) (*P* < 0.0001) as were the ratios of IL-23/IL-4, IL-23/IL-10, and IL-23/IL-13 (*P* < 0.03, *P* < 0.01, and *P* < 0.03, resp.).

### 3.4. Comparison of Cytokine Profiles in IUGR with and without Placental Insufficiency

The proinflammatory Th1-inducing cytokine IL-12 is produced at higher levels in IUGR with placental insufficiency (29 pg/mL ± 3.3) than in IUGR without placental insufficiency (12 pg/mL ± 2.1) (*P* < 0.01). On the contrary, the anti-inflammatory Th2 cytokine IL-10 is produced at lower levels in IUGR with placental insufficiency (240 pg/mL ± 29) as compared to IUGR without placental insufficiency (421 pg/mL ± 55) (*P* < 0.01). None of the other cytokines are significantly different. Three of the proinflammatory : anti-inflammatory cytokine ratios are higher in IUGR with placental insufficiency; these are IL-12/IL-10 (*P* < 0.005), IL-12/IL-4 (*P* < 0.02), and IL-8/IL-10 (*P* < 0.01). We infer from this data that a stronger proinflammatory cytokine bias exists in IUGR with placental insufficiency as compared to IUGR without placental insufficiency.

## 4. Discussion

This study was undertaken with the expectation that studies of this sort may lead to the identification of immunologic etiologies of fetal growth restriction or to immune-mediated pathophysiologic mechanisms that could lead to fetal growth restriction even if the initial etiology is nonimmunologic. While previous studies have reported the estimation of cytokine levels in the serum of women with IUGR, this is the first to present data on cytokine production profiles of maternal lymphocytes after stimulation with trophoblast antigens. T lymphocytes can be activated with mitogen, anti-CD3, and with antigens; in this study we chose to stimulate maternal T lymphocytes in PBMC with trophoblast antigens. The trophoblast cell line, JEG-3, used to prepare a trophoblast antigen extract has characteristics similar to early normal human trophoblast cells, including invasive characteristics, endocrine, and antigenic features. Previous studies using antigen extracts from this cell line [20–22] demonstrated higher Th1-type reactivity and lower Th2-type to trophoblast antigens in women with unexplained recurrent miscarriage as compared to women with a history of normal pregnancy.

We found interesting differences in the levels of some pro- and anti-inflammatory cytokines between IUGR and normal pregnancy and between IUGR with and without placental insufficiency.

The proinflammatory chemotactic cytokine IL-8 is consistently produced at significantly higher levels in IUGR subjects as a group when compared to normal pregnancy, and also in IUGR with placental insufficiency and IUGR without placental insufficiency as compared to normal pregnancy. IL-8 is induced by a variety of stimuli that include lipopolysaccharide, live bacteria, and other proinflammatory cytokines such as TNF and IL-1 [25] and it, in turn, induces chemotaxis of inflammatory cells. It is the principal recruiter of neutrophils, the signature cell of acute inflammatory responses. In addition to recruiting cells to the site of inflammation, IL-8 also retains cells once they have arrived and stimulates neutrophils to a higher state of activation [25]. IL-8 is relatively unique in that it is produced early in the inflammatory response but persists for a prolonged period of time, unlike other proinflammatory cytokines that are usually made and cleared in a matter of hours in vivo. IL-8 is thus a key inducer and sustainer of local tissue inflammation [26].

Increased maternal and umbilical cord serum levels of IL-8 were recently shown to be higher in pre-eclampsia complicated by IUGR than in pre-eclampsia with normal fetal growth [27, 28]. However, this was not reflected in a study by Johnson et al. [29] who found no differences in the levels of IL-8 in IUGR versus normal pregnancy. Hahn-Zoric et al. [30] found higher placental levels of IL-8 in IUGR compared with appropriately developed neonates. It has been suggested that local action of cytokines like IL-8 may be responsible for the increased infiltration of macrophages that are seen in IUGR, and activated macrophages could contribute to placental dysfunction [31].

In addition to the higher production of IL-8 by PBMC from women with IUGR, the proinflammatory cytokines IFN*γ* and TNF*α* are also produced at higher levels in IUGR with placental insufficiency versus normal pregnancy. IFN*γ* and TNF*α* are the prime culprits in the development of chronic inflammation [32] and both of them are cytotoxic cytokines that induce apoptosis of target cells. IFN*γ*, a classical Th1 cytokine, is a crucial inducer of Th1 development and affects the activation and function of a variety of cells that include T cells, B cells, macrophages, and NK cells. TNF*α* is one of the most prominent inflammatory mediators and initiates inflammatory reactions of the innate immune system, including the induction of cytokine production, activation, and expression of adhesion molecules and thrombosis [33]. Along with IL-1 and IL-6, TNF*α* induces many of the localized changes seen in acute inflammatory reactions such as increased vascular permeability, induction of chemokine production, and the expression of adhesion molecules on vascular endothelia. 

Neta et al. [34] reported that lower levels of IFN*γ* were associated with a reduced risk of small-for-gestational age babies and suggest that lower levels of IFN*γ* could indicate impairment of trophoblast function leading the authors to support a protective role for IFN*γ*. This is in contrast to our observation that IFN*γ* is produced at higher concentrations by PBMC from women with IUGR with placental insufficiency. 

While there appear to be differences in observations on IFN*γ* in IUGR, evidence for an association between TNF*α* and IUGR seems to be compelling. Increased levels of TNF*α* have been reported in the serum of pregnancies complicated with IUGR [35]. Amarilyo et al. [36] showed higher levels of TNF*α* in the cord blood of IUGR infants and suggest that a state of inflammation exists in such infants. TNF*α* levels in maternal and umbilical cord serum are reported to be higher in pre-eclampsia complicated by IUGR than in pre-eclampsia with normal fetal growth [25]. Holcberg et al. [37] found that increased TNF secretion in placentas of IUGR fetuses is related to enhanced vasoconstriction of the fetal placental vascular bed, and Rogerson et al. [38] reported that placental TNF*α* levels are increased in low birth weight infants associated with malaria. 

Overproduction of TNF*α* and other proinflammatory cytokines has been proposed to be important in the development of fetal growth restriction in response to hypoxia [39], possibly by decreasing amino acid uptake by the fetus [40]. TNF*α* has other effects on the placenta that may be relevant; it inhibits the growth of the trophoblast [41], interferes with placental development and invasion of the spiral arteries, is directly toxic to endothelium, and may damage the decidual vasculature [42]. TNF interferes with the anticoagulant system and may induce placental thrombosis [43]. Holcberg et al. [37] found that increased TNF secretion in placentas of IUGR fetuses is related to enhanced vasoconstriction of the fetal placental vascular bed. 

Perhaps the most likely mechanism by which TNF*α* may contribute to IUGR is by causing apoptosis of trophoblast cells. Trophoblast cells of pregnancies with IUGR are more sensitive to apoptosis in response to cytokines and hypoxia when compared to trophoblast cells from normal pregnancies, and it is speculated that this dysregulated apoptosis may lead to the placental dysfunction seen in IUGR [44]. The apoptotic effect of TNF*α* is well known; it has been shown to kill trophoblast cells [45], and it is likely that the increased apoptosis in IUGR is due in part to cytokines like TNF*α* [46]. In fact, IUGR has been shown to be characterized by enhanced trophoblast apoptosis, and this has been suggested to lead to abnormal placentation, inadequate spiral artery remodelling, and uteroplacental vascular insufficiency [47].

If proinflammatory cytokines, such as IL-8 and TNF*α*, pose the risk of adverse outcomes of pregnancy, presumably these may have to be countered by anti-inflammatory cytokines. Indeed, the levels of the anti-inflammatory cytokine IL-13 are higher in normal pregnancy as compared to the IUGR group and to IUGR without placental insufficiency; we also observed a trend towards lower IL-13 levels in IUGR with placental insufficiency (*P* < 0.059). IL-13 is a Th2 cytokine with anti-inflammatory properties. IL-13 inhibits the production of the inflammatory cytokines IL-6, IL-12, TNF*α*, and IL-8, prevents pathological inflammation at mucosal surfaces, and inhibits cytotoxicity [48]. The enhanced levels of IL-13 in normal pregnancy versus IUGR may reflect a stronger Th2 bias or an anti-inflammatory cytokine bias in normal pregnancy. Further, Dealtry et al. [49] demonstrated the expression of IL-13 by human trophoblast cells and suggest that IL-13 may play important roles in maternal-fetal dialogue that aids in the establishment and maintenance of the placenta. Thus, the decreased levels of IL-13 production in IUGR observed in this study may be pertinent.

In addition to the proinflammatory bias in IUGR suggested by elevated levels of IL-8 and decreased levels of IL-13, a comparison of pro- to anti-inflammatory cytokines is also interesting. Ratios of IL-6/IL-13, IL-8/IL-13, IL-12/IL-13, and TNF*α*/IL-13 are all significantly higher in the IUGR group compared to normal pregnancy (Table 2). The IL-12/IL-13 and IL-12/IL-10 ratios are higher in IUGR with placental insufficiency, also suggestive of a stronger inflammatory skew in IUGR with placental insufficiency.

Pregnancy has been suggested to bring about a mild state of inflammation [50], and Li and Huang [31] speculate that exaggerated or excessive inflammation could result in adverse outcomes such as IUGR via a vicious cycle of coagulation, thrombosis, and inflammation. Thus, mutually enhancing cascades of coagulation and inflammation may be part of the etiopathogenesis of IUGR. 

One of the objectives of this study was to compare IUGR with and without placental insufficiency. IUGR without placental insufficiency is, generally, due to constitutional causes in the absence of obvious placental pathologies, while IUGR with placental insufficiency manifests with significant placental pathology and decreased maternal-fetal blood flow. This led us to speculate that IUGR pregnancies with placental insufficiency may have a predominant proinflammatory cytokine bias. Our data suggests that this might indeed be the case. IL-12 levels are significantly higher in IUGR with placental insufficiency (Figure 2), while IL-10 levels are significantly lower (Figure 1). Three of the ratios are also higher in IUGR without placental insufficiency: IL-12/IL-10, IL-12/IL-4, and IL-8/IL-10. None of the other cytokine ratios were significantly different between the two subgroups. While this study should have ideally included cases of non-IUGR with placental insufficiency, our data suggests that there is a stronger tilt towards proinflammatory cytokines in IUGR *with* placental insufficiency than in IUGR *without* placental insufficiency. 

The lower-level of IL-10 in IUGR with placental insufficiency is interesting as it is perhaps the most important anti-inflammatory cytokine found within the human immune response. It inhibits Th1 cytokine release, NF-*κ*B signaling, expression of HLA class II molecules, macrophage, and dendritic cell function [51]. As IL-10 has profound anti-inflammatory properties, the decreased levels of IL-10 in IUGR with placental insufficiency, may be indicative of a lower proinflammatory bias in this subgroup versus IUGR without placental insufficiency subgroup. Previous studies have shown decreased levels of IL-10 in the placentas of IUGR pregnancies and this has been suggested to be relevant to the pathogenesis of IUGR [30]. Given its ability to inhibit the synthesis of proinflammatory cytokines and macrophage activity and its role in reducing apoptosis [52], IL-10 may, in part, be responsible for the maintenance of a balance against a proinflammatory bias in normal pregnancy.

IL-23 levels in this study present an interesting conundrum; we found significantly higher-levels of IL-23 in normal pregnancy as compared to the three IUGR groups in trophoblast antigen-stimulated cultures. IL-23 is known to have many similarities to IL-12. Along with IL-12, IL-23 plays an important role in bridging innate and acquired immune responses and causes multiorgan inflammation with elevated expression of inflammatory cytokines like TNF*α* and IL-1 [53]. It is not immediately apparent how lower levels of IL-23 are related to the pathogenesis of IUGR, but there are a few interesting leads. IL-23 is not required for Th1 responses and it appears to act not via the Th1 pathway but along the IL-23/IL-17 pathway of inflammatory responses; in fact the addition of IL-23 to murine T-cell cultures pushes Th development away from Th1/IFN*γ* differentiation [53]. Remarkably enough, IL-23 has been proposed to actually offer protection against the deleterious effects of TNF in implantation, explaining embryo survival in a TNF-rich environment [54]. Also, Vujisić et al. [55] reported significantly higher levels of IL-23 in the follicular fluid taken from follicles containing oocytes, when compared with those without an oocyte; these authors propose that increased concentrations of IL-23 in follicles containing oocytes may indicate a beneficial role for this cytokine in reproduction. Our observation of lower levels of IL-23 in IUGR samples seems to support the idea of a beneficial role for IL-23 in normal pregnancy.

Based on the Th1 shift reported in recurrent miscarriage, preterm labor, and pre-eclampsia, our initial premise was to ascertain whether a similar Th1 bias exists in IUGR. In a murine model of fetal growth restriction, induced by Porphyromonas gingivalis infection, Lin et al. [56] showed this bacterium adversely affects normal fetal development via direct placental invasion and induction of fetus-specific placental immune responses characterized by a proinflammatory Th1-type cytokine profile. They found that mRNA levels of IFN*γ* and IL-2 were significantly increased in placentas of fetuses with growth restriction, while expression of IL-10 was significantly decreased in the same group. The authors concluded that fetal growth restriction in this model is associated with a shift in the placental Th1/Th2 cytokine balance. We do not observe an obvious Th1/Th2 bias in the cytokine production profiles of maternal PBMC; so we suggest that it is more likely that a general proinflammatory, rather than a more specific Th1-bias, operates in IUGR. This contention is based on the lack of a predominance of Th1/Th2 cytokines such as IFN*γ* and IL-4. However, our comparison of cytokine profiles in IUGR with and without placental insufficiency showed elevated production of IL-12 and decreased production in IUGR with placental insufficiency; IL-12 is a Th1-inducing cytokine, while IL-10 is a Th2-type cytokine and it is tempting to suggest the possibility of a Th1-bias in IUGR with placental insufficiency when compared to IUGR without placental insufficiency.

## 5. Conclusions

IUGR is a serious obstetric problem and it is important that its etiologies and pathogenetic mechanisms be elucidated. Identifying possible associations between IUGR and immunological effectors such as cytokines will help us understand the pathophysiology of this disease and define markers that can predict this condition. Understanding immunological mechanisms of normal pregnancy and of complications such as IUGR could lead to the development of regimens to improve fetal growth and development.

This study suggests that a proinflammatory cytokine bias exists in maternal peripheral blood mononuclear cells of women with IUGR when compared to normal pregnancy. It also supports the notion of a stronger proinflammatory tilt in IUGR with placental insufficiency as compared to IUGR without placental insufficiency. This conclusion is based on levels of cytokines produced by maternal peripheral blood cells as well as calculated ratios of pro- to anti-inflammatory cytokines. Future research should enable the elucidation of the roles of cytokines in the pathophysiology of IUGR as well as the development of new therapies that will aid the management of this condition.

## Figures and Tables

**Table 1:** Demographic data on subjects in this study.

	Control *N* = 22	IUGR *N* = 36	*P* value	IUGR with placental Insufficiency *N* = 19	IUGR without placental Insufficiency *N* = 17	*P* value
Maternal age	32.4 ± 4.2	35.1 ± 3.7	NS	34.6 ± 3.3	36.1 ± 4.3	NS
Mode of delivery						
C.S.	6	15	—	9	6	—
S.V.D	16	21		10	11	
Outcome						
Preterm	2	8	—	6	2	—
Term	20	28		13	15	
Birthweight (Kg)	3.6 ± 1.2	2.3 ± 0.7	<0.001	2.0 ± 0.9	1.9 ± 0.6	NS

NS: Nonsignificant; C.S.: Caesarian section; S.V.D.: Single vaginal delivery.

**Table 2:** Means of ratios of proinflammatory to anti-inflammatory cytokines. All possible combinations of pro- and anti-inflammatory cytokines were compared, but only the ones which are significantly different are presented in this table. *I* > *N* indicates that the ratio is higher in IUGR than in normal pregnancy, *WO* > *N* indicates that the ratio in IUGR without placental insufficiency subgroup is higher than in normal pregnancy group, and so on.

Cytokine ratio	Normal pregnancy control (*N*)	Total IUGR (*I*)	IUGR with placental insufficiency (*W*)	IUGR without placental insufficiency (*WO*)	Significant differences
IL-6/IL-13	3265	18134	7142	22189	*I* *> N * *WO > N *

IL-8/IL-10	6	7	9	4	*W > WO*

IL-8/IL-13	197	1169	526	1285	*I > N* *WO > N*

IL-12/IL-4	14	17	26	10	*W > WO*

IL-12/IL-10	0.04	0.08	0.13	0.04	*W > N* *W > WO*

IL-12/IL-13	1.4	6	4	8	*I > N* *W > N*

IFN*γ*/IL-10	0.14	0.23	0.44	0.09	*N > WO*

TNF*α*/IL-13	11	210	91	101	*I > N*

IL-23/IL-4	357	248	210	273	*N > I* *C > W* *N > WO*

IL-23/IL-10	23	1.4	1.7	1.2	*N > I* *N > WO*

IL-23/IL-13	58	230	99	238	*N > WO*

## Appendix

The nine ELISA kits were specific for the target human cytokine with no cross-reactivity or interference with other cytokines or cytokine receptors.
The IL-4 kit has a sensitivity of 5 pg/mL. Coefficient of variation (CV) for intra-assay precision ranged between 2.1 and 5.6%, while interassay CVs ranged from 4.8% to 9.7%.
The IL-6 kit has a sensitivity of 3 pg/mL, intra-assay CVs ranged between 1.6% and 6.8%, while interassay CVs ranged from 7.9% to 11.6%.
The sensitivity of the IL-8 kit is 8 pg/mL; the intra-assay CVs were between 2.3% and 5.5% and interassay CVs ranged from 7.6% to 10.1%.
IL-10 was measured at a sensitivity of 5 pg/mL, intra-assay CVs ranged between 3.3% and 4%, while interassay CVs were between 5.6% and 8.6%.
The IL-12 ELISA kit measures the IL-12 heterodimer, has an intra-assay CV of 5.5% and interassay CV of 10%.
The sensitivity of the IL-13 ELISA kit is 0.7 pg/mL, intra- and interassay CVs are 6% and 4.6%, respectively.
The human IL-23 Platinum ELISA measures the p19 subunit and has a sensitivity of 4 pg/mL, intra-assay CV is 5.9%, interassay CV is 6.3%.
IFN*γ* was measured at a sensitivity of 0.08 IU/mL, intra-assay CVs ranged between 2.2% and 12.6% and interassay CVs ranged from 6.2% to 12.2%.
The sensitivity of the TNF*α* kit is 5 pg/mL; intra-assay CVs for this kit were between 1.6% and 10% while interassay CVs were between 5.4% and 12.8%.
